# Emotion dysregulation in adults suffering from attention deficit hyperactivity disorder (ADHD), a comparison with borderline personality disorder (BPD)

**DOI:** 10.1186/s40479-019-0108-1

**Published:** 2019-07-18

**Authors:** Eva Rüfenacht, Sebastian Euler, Paco Prada, Rosetta Nicastro, Karen Dieben, Roland Hasler, Eléonore Pham, Nader Perroud, Sébastien Weibel

**Affiliations:** 1 TRE Unit, Department of Psychiatry, Division of Psychiatric Specialties, Department of Mental Health and Psychiatry, University Hospitals of Geneva, 20bis rue de Lausanne, Geneva, Switzerland; 2 Department of Consultation Psychiatry and Psychosomatics, University Hospital Zurich and University of Zurich, Zurich, Switzerland; 3 Department of Psychiatry, Faculty of Medicine, University of Geneva, Geneva, Switzerland; 4 Pôle de Psychiatrie, Santé Mentale et Addictologie, University Hospital Strasbourg, Strasbourg, France; 5 INSERM 1114, Strasbourg, France; 6 FMTS, University of Strasbourg, Strasbourg, France

**Keywords:** Emotional dysregulation, Attention deficit hyperactivity disorder, Borderline personality disorder, Cognitive emotion regulation strategies, Comorbid ADHD and borderline personality disorder, Metacognitive abilities, Emotional perception

## Abstract

**Background:**

Emotional dysregulation (ED) is now considered as an important symptom of attention deficit hyperactivity disorder (ADHD). It is believed to have a considerable impact on the severity of the disorder, one’s global functioning, and the prognosis. Our research aimed to evaluate and compare ED and cognitive emotional regulation strategies between ADHD and borderline personality disorder (BPD) patients.

**Methods:**

Four hundred six French-speaking outpatients (*N* = 279 ADHD, *N* = 70 BPD, *N* = 60 BPD + ADHD) were assessed with the Emotion Reactivity Scale (ERS), the Cognitive Emotional Regulation Questionnaire (CERQ), The Basic Empathy Scale (BES-A), the Adult ADHD Self-Report Scale (ARSV-v1.1) and the Beck Depression Inventory II (BDI-II). ADHD, BPD and comorbid patients were compared with each other and with samples of controls extracted from already published data.

**Results:**

ADHD patients, although having higher ED than samples derived from the general population, had less ED, better control over their emotions with higher use of adaptive cognitive strategies and lesser use of non-adaptive strategies than BPD patients. However, ADHD subjects had similar scores as BPD subjects when looking at difficulties in perceiving self and others. ED generated considerable distress in all groups and was also positively associated with ADHD symptomatology. ADHD patients with comorbid BPD had the highest scores of ED.

**Conclusions:**

Our results suggest that there may be similarly inefficient cognitive emotional regulation skills leading to ED in both disorders (ADHD and BPD). However, ADHD patients showed a higher use of adaptive cognitive emotional strategies and a lower level of ED than BPD patients.

## Background

There is increasing evidence of a close link between emotional symptoms and attention deficit hyperactivity disorder (ADHD) [1–3]. Several studies suggest that besides attentional and hyperactive-impulsive symptoms, emotional dysregulation (ED) is a core symptom of ADHD, both in childhood and adulthood [4–6].

Studies have estimated that 34 to 70% of adults suffering from ADHD have difficulties regulating their emotions [4–6]. These emotional symptoms have been associated with poor global functioning including lower quality of life, interpersonal and professional difficulties, poor prognosis, and severity of attentional and hyperactive-impulsive symptoms in adults as well as in children [1, 4, 7–9]. Research has shown that ED in ADHD is similar to the nature of ED found in borderline personality disorder (BPD), comprising of increased instability and intensity of negative emotions and a slow return to emotional baseline when activated [4, 10]. ED in BPD is considered a fundamental mechanism of the disorder, and this dimension concerns at least two of the DSM-5 criteria for the disorder. This makes BPD the “gold standard” disorder to which other disorders (such as ADHD) can be compared to assess their level of ED. The frequent comorbidity between ADHD and BPD (around one third of BPD patients have ADHD, and vice versa) suggests common mechanisms and aetiology [11, 12]. There is some evidence that ED symptoms mediate the relationship between retrospectively assessed childhood ADHD and adulthood BPD symptoms [13]. Additionally, ADHD has been identified as a potential risk factor for adulthood BPD development [14]. Yet few studies directly compared ED in BPD and ADHD and none considered comorbid ADHD+BPD patients [14–16].

Besides the usual description of ED in BPD, namely heightened emotional reactivity to environmental stimuli, inadequate cognitive emotion regulation strategies resulting form difficulty identifying, differentiating, and labeling emotions have also been suggested as important components of ED [17–22]. BPD patients indeed engage in more maladaptive cognitive emotion regulation strategies such as ‘thought suppression’ (efforts to suppress emotional response to a subjective experience) and ‘rumination’ (disproportionate focus on emotional experiences) and show fewer adaptive strategies such as ‘cognitive reappraisal’ (have a different perspective on a situation to change one-self feeling) or ‘acceptance’ (endorse a nonjudgmental stance toward internal experiences) compared to healthy individuals [18–22]. The use of more maladaptive cognitive strategies has been associated, in BPD, with higher severity of the disorder (more harmful behaviours) [18]. Finally, poor mentalizing capacities (the process sustaining our understanding of human action as driven by mental states including, among other dimensions, empathy) are also considered to underlie ED in BPD. Indeed, poor reflective functioning, the operationalized measure of mentalizing capacity, has been directly linked to poor emotion regulation in BPD [14].

Although several hypotheses explaining ED in ADHD have been suggested, including executive function deficits, social cognitions deficit, impaired emotional perception/processing, lack of empathy and impairment in first and second order theory of mind and poor mentalizing capacities [14, 23–31], less is known about the efficiency of cognitive emotion regulation strategies used by ADHD patients to cope with emotional outbursts. Some studies on adults suffering from ADHD have associated ED and poor emotional regulation strategies such as less use of ‘cognitive reappraisal’ and more use of ‘suppression’ [32]. Poor cognitive emotion regulation skills in ADHD have also been linked to impaired metacognitive abilities, including difficulties to focus oneself, and a diminished ability to re-evaluate goals and behaviours, prompting poor situation evaluations, thereby magnifying the associated emotional state [3, 4, 24, 32]. A better understanding of ED, cognitive emotion regulation strategies and their correlation with ADHD disorder severity is clearly needed.

The aim of our study was therefore to compare emotional reactivity, cognitive emotional regulation strategies, and empathy in subjects suffering of ADHD to subjects with BPD, or comorbid ADHD and BPD. We also evaluated the relationship between ADHD symptoms and ED severity.

## Methods

### Participants and procedure

Four hundred and six French-speaking outpatients (*N* = 279 ADHD, *N* = 70 BPD and *N* = 60 BPD + ADHD) were recruited in a specialized centre for diagnosis and treatment of adults suffering from ADHD and BPD at the University Hospitals of Geneva.

Patients underwent a clinical evaluation at their entry of the program conducted by trained psychiatrists to ascertain the diagnosis of BPD and/or ADHD according to DSM-5 criteria, and to exclude any organic condition and/or comorbid disorders that could better explain the symptoms. With the exception of ADHD and BPD (see below), other comorbidities were assessed clinically and using medical records only, and no structured interviews were used. In addition, subjects were administered the *Adult ADHD Self-Report Scale-Version 1.1 part A and B* (ASRS v1.1) [33]; and the *Borderline Symptoms Check-List* (BSL-23) for BPD patients as previously described [34]. Finally, ADHD diagnosis was confirmed by the structured *Diagnostic Interview for ADHD in adults* (DIVA 2.0), assessing DSM-IV ADHD criteria [35] (but DSM-5 criteria were applied). BPD diagnosis was confirmed by the structured diagnostic *Screening Interview for Axis II disorders* (SCID-II), assessing DSM-IV BPD criteria [36].

Of note, most of the ADHD patients were free of ADHD medication when participating to the current study. Indeed, most of them were not diagnosed previously and underwent the clinical and structured evaluations (including the self-report questionnaires described below) when psychostimulants were not yet started.

All participants completed the *Beck Depression Inventory II* (BDI-II) to assess the current level of depression as an indirect tool to evaluate the current distress associated with suffering from ADHD or BPD [37]. BDI-II has been shown to be a good proxy to assess subjective distress associated with ADHD [32, 38].

The study was approved by the ethics committee of University Hospitals of Geneva and all subjects provided informed written consent.

### Assessment instruments used in the study

Several instruments were used to assess emotion regulation and reactivity, cognitive strategies to avoid worries, and empathy.

The *Emotion Reactivity Scale* (ERS) is a self-report questionnaire enquiring about emotional experience on regular basis. It consists of 21 items measuring emotion reactivity, based on three aspects: emotion sensitivity, intensity, and persistence. Each item is rated on a 5-point Likert scale from 0 (not at all like me) to 4 (completely like me), with scores ranging from 0 to 40 for emotion sensitivity, 0 to 28 for emotion intensity and 0 to 16 for persistence, and total scores ranging from 0 to 84 [39]. The validated French version was used [40]. Studies found that total scale and subscales had good internal consistency, and factor analyses revealed that both a single-factor and a three-factor model fit the data well [40, 41]. The scale can therefore be used with total scores and subscores.

The *Cognitive Emotional Regulation Questionnaire* (CERQ) is a 36-item questionnaire consisting of 9 conceptually different subscales based on different cognitive emotion regulation strategies, overall divided in two main domains: the *adaptive strategies* domain entails putting into perspective, positive refocusing, positive reappraisal, acceptance, refocus on planning; the *non-adaptive strategies* domain comprises self-blame, other-blame, rumination, and catastrophizing. Each subscale contains 4 items referring to thoughts after the experience of a threatening or stressful life event. The items are measured on a 5-point Likert scale, ranging from 1 (almost never) to 5 (almost always) [42]. The validated French version was used [43]. Exploratory and confirmatory factor analyses showed that a nine-factor model explained the data in the original and the French version [42, 43].

The *Basic Empathy Scale in Adults* (BES-A) is a 20-item self-report scale, focusing on two components of empathy, the cognitive and affective components [44, 45]. Each item is rated on a 5-point Likert scale (1 = Strongly Disagree to 5 = Strongly Agree; 7 reversed items), with a score ranging from 20 (deficit in empathy) to 100 (high level of empathy). Nine items evaluate cognitive empathy and 11 affective empathy. The validated French version was used [46]. The scale, initially validated in adolescents, was subsequently validated in an adult sample [45] showing that the two-factor model was appropriate.

### Sample of controls

ADHD patients were compared to a sample of controls extracted from published data that have used the ERS, the CERQ or the BES-A [18, 39, 40, 45, 47, 48]. Of note, controls were not matched for age or gender.

### Statistics

All analyses were performed using Stata v14. Univariate comparisons between clinical groups were conducted using the chi square test for qualitative variables (gender, comorbidity, treatment) or Fisher’s exact test when assumption of frequencies for chi square test were not met, and a one-way ANOVA for quantitative variables (age, clinical scales). T-tests were used to compare ADHD patients to a sample of controls extracted from published data that have used the ERS, the CERQ or the BES-A [18, 39, 40, 45, 47, 48]. Statistical significance was accepted for *p* < 0.05. Post-hoc tests were performed using pairwise comparisons (Bonferroni correction).

Questionnaire scores were analyzed with linear regression models, using the diagnostic group as a fixed variable. Models were adjusted for age and gender in the event of significant differences between groups. For all continuous predictors, we examined the assumption of normality of the distribution of residuals with residual plots. These revealed no breach of model assumptions.

Secondary analyses were performed to assess the relation between symptomatology and questionnaire scores. ASRS total score (for ADHD and ADHD+BPD groups) or BDI-II scores were added into the model as continuous predictors. Finally, association with ADHD presentation and current depressive episode were also assessed.

## Results

### Demographic and clinical characteristics

ADHD patients were older than the other groups (*F* = 6.5; df = 2/405; *p* = 0.002). BPD and BPD + ADHD patients were more likely to be female than ADHD patients (X2 = 89.7; *p* < 0.001). ADHD patients were more likely to have a job than the two other groups (X2 = 9.21; *p* = 0.01) and to have 2 or more children (X2 = 10.11; *p* = 0.04) (Table 1).

**Table 1:** Clinical and demographic characteristics of ADHD, BPD and comorbid ADHD+BPD patients

	ADHD *N* = 279		BPD *N* = 70		ADHD+BPD *N* = 60		*P*
	mean or N	sd or %	mean or N	sd or %	mean or N	sd or %	
Age	35.49	12.86	31.66	9.08	30.29	8.64	0.002
Gender							
Female	122	43.73	66	94.29	55	91.67	< 0.001
Civil status^a^							
Single (vs not single)	130	49.24	36	51.43	33	57.89	0.493
Children^a^							
0	163	61.74	46	65.71	43	74.14	0.04
1	28	10.61	13	18.57	7	12.07	
≥ 2	73	27.65	11	15.71	8	13.79	
Job^a^							
Yes (vs no)	157	61.33	30	42.86	26	48.15	0.01
Years of education^a^	15.52	2.86	15.23	3.18	14.78	2.69	0.236
Lifetime comorbidities							
Major depressive disorder	122	43.73	58	82.86	39	65.00	< 0.001
Bipolar disorder	6	2.15	7	10	6	10.00	0.002
Anxiety disorder^b^	57	20.43	58	82.86	33	55.00	< 0.001
Eating Disorders	10	3.58	14	20	9	15.00	< 0.001
Substance use disorder	70	25.09	24	34.29	24	40.00	0.03
ADHD type							
Attentional	109	39.07	–	–	27	46.55	0.508
Hyperactive-impulsive	12	4.30	–	–	3	5.17	
Mixte	158	56.63	–	–	28	48.28	
Medication							
Psychostimulant	36	12.90	1	1.43	7	11.66	0.02
Antidepressant	57	20.43	36	51.43	24	40.00	< 0.001
Antipsychotic	12	4.30	11	15.71	8	13.33	0.001
Benzodiazepine	28	10.03	20	28.57	10	16.66	< 0.001
Mood stabilizer	2	0.72	7	10.00	4	6.66	< 0.001
ASRS v1.1							
Inat.	25.23	5.37	22.33	6.38	25.65	5.56	< 0.001
Hyper.	21.23	6.87	19.92	6.49	23.88	7.52	< 0.001
Total	46.47	10.18	42.25	11.31	49.53	10.87	< 0.001
BDI-II	20.79	11.99	34.75	11.65	33.19	9.78	< 0.001

Means, absolute numbers (N), SD, % and comparisons (*p*-value) between ADHD, BPD, and comorbid ADHD+BPD groups for clinical and demographic characteristics

^a^ missing values encountered, ^b^Including: generalized anxiety disorder, panic disorder, social phobia and obsessive-compulsive disorder

Patients with ADHD were less likely to have other comorbid disorders than BPD and ADHD+BPD patients. Taken together 63.44% (*N* = 177/279) of ADHD patients did have at least one comorbidity compared to 97.14% (*N* = 68/70) of BPD patients and 93.33% (*N* = 56/60) of the ADHD+BPD patients. The use of medication differed among participants. A small fraction of patients took stimulants: 12.90% of ADHD patients, 11.66% of ADHD+BPD patients, and 1.43% of BPD patients. No patients took non-stimulant ADHD medication. The use of other medications (antipsychotics, antidepressants, benzodiazepines, mood stabilizers) were lower in ADHD patients compared to the two other groups (Table 1). The groups also differed in terms of symptomatology. ADHD symptoms as measured by the ASRS v1.1 were the highest in the BPD + ADHD group, and the lowest in BPD (*F* = 7.87; df = 2/403; *p* < 0.001). BDI-II scores were higher in BPD and BPD + ADHD than in ADHD group (*F* = 55.79; df = 2/398; *p* < 0.001) (Table 1).

### Emotional reactivity scale

ERS total and subscale scores are displayed in Table 2. BPD and BPD + ADHD patients scored higher than ADHD patients in ERS total and in each subscale: sensibility, intensity and persistence (each *p* < 0.001) (Table 2). BPD and BPD + ADHD did not differ.

**Table 2:** Comparisons of ERS, CERQ and BES results between ADHD, BPD and comorbid ADHD+BPD

		ADHD		BPD		vs. *ADHD*		ADHD+BPD		vs. *ADHD*		vs. *BPD*	
		mean	sd	mean	sd	*coeff.*	*p*	mean	sd	*coeff.*	*p*	*coeff.*	*p*
ERS	Sensitivity	22.31	8.78	29.23	7.77	4.82	< 0.001	31.26	5.95	6.97	< 0.001	2.14	0.14
	Arousal/Intensity	15.84	7.06	22.07	5.65	4.32	< 0.001	22.86	4.49	5.22	< 0.001	0.90	0.42
	Persistence	9.23	3.78	11.71	3.26	1.62	< 0.001	12.50	2.70	2.46	< 0.001	0.84	0.18
	Total	47.38	18.39	63.01	15.61	10.76	< 0.001	66.61	12.10	14.64	< 0.001	3.88	0.19
CERQ	Self-Blame	11.53	3.75	13.82	3.56	1.72	< 0.005	14.06	3.60	1.96	< 0.001	0.24	0.72
	Acceptance	12.83	3.38	13.12	3.46	0.71	0.153	13.38	3.44	0.93	0.08	0.22	0.72
	Rumination	12.94	3.86	14.36	3.87	0.56	0.301	14.79	3.54	1.03	0.084	0.45	0.50
	Positive Refocusing	9.00	3.68	7.71	3.63	−1.29	< 0.05	7.39	3.17	−1.65	< 0.005	−0.36	0.58
	Refocusing on Planning	12.94	3.63	10.82	3.93	−2.32	< 0.001	11.65	3.53	−1.47	< 0.05	0.85	0.19
	Positive Reapraisial	12.45	3.89	10.07	4.12	−2.21	< 0.001	10.53	3.56	−1.75	< 0.005	0.46	0.67
	Putting into perspective	12.27	3.82	10.76	3.83	− 1.44	< 0.05	10.81	3.66	−1.40	< 0.05	0.05	0.95
	Catastrophizing	8.66	3.60	10.27	3.86	1.26	< 0.05	10.79	3.93	1.73	< 0.005	0.47	0.47
	Blaming others	8.84	3.53	9.49	3.88	0.49	0.358	10.61	3.60	1.58	< 0.005	1.09	0.09
	Adaptive Strategies Tot.	59.49	13.29	52.58	14.02	−6.44	< 0.001	53.76	11.86	−5.32	< 0.05	1.12	0.64
	Non-adaptive Strategies Tot.	41.97	10.66	47.93	10.80	4.04	< 0.01	50.57	10.32	6.62	< 0.001	2.58	0.17
BES	Cognitive	32.39	3.86	33.15	4.56	−0.04	9.44	31.79	4.60	−1.35	< 0.05	−1.31	0.07
	Affective	39.81	7.37	42.93	7.34	0.44	0.658	42.74	6.09	0.41	0.697	−0.03	0.98
	Total	72.21	9.82	76.07	10.46	0.40	0.771	74.53	9.11	−0.94	0.517	−1.34	0.42

Comparisons adjusted on age and gender

Means, SD and comparisons (coefficient and *p* value) between ADHD, BPD and comorbid ADHD+BPD for Emotion Regulation Scale (ERS), Cognitive Emotion Regulation Questionnaire (CERQ) and Basic Empathy Scale (BES)

Our ADHD patients displayed higher ED as measured by the ERS total score compared to a patient population recruited from community and local psychiatric clinics (t = 4.79; *p* < 0.001; Mean: 36.66 standard deviation (SD): 17.52 vs Mean: 47.38 SD: 18.39) [39] as well as compared to French-speaking participants from the community (t = 8.03; *p* < 0.001; Mean: 35.02 SD: 17.14) [40] (Table 3).

**Table 3:** Comparisons of ERS, CERQ and BES results between ADHD and control samples

		ADHD		Controls				Controls			
		*N* = 279		*N* = 87^a^		t	*p*	*N* = 258^b^		t	*p*
		mean	sd	mean	sd			mean	sd		
ERS	Sensitivity	22.31	8.78	16.29	8.61	5.61	< 0.001	16.43	8.37	7.93	< 0.001
	Arousal/Intensity	15.84	7.06	13.11	6.3	3.22	< 0.001	11.48	6.33	7.51	< 0.001
	Persistence	9.23	3.78	7.26	4.03	4.18	< 0.001	7.11	3.47	6.75	< 0–001
	Total	47.38	18.39	36.66	17.52	4.79	< 0.001	35.02	17.14	8.03	< 0.001
		*N* = 279		*N* = 611^c^		t	*p*	*n* = 32^d^		t	*p*
		mean	sd	mean	sd			mean	sd		
CERQ	Self-Blame	11.53	3.75	8.22	2.96	14.19	< 0.001	–	–	–	
	Acceptance	12.83	3.38	11.01	3.53	7.23	< 0.001	12.41	3.16	0.67	0.503
	Rumination	12.94	3.86	10.46	3.72	9.12	< 0.001	–	–	–	
	Positive Refocusing	9	3.68	10.01	3.53	−3.91	< 0.001	9.5	3.81	−0.73	0.469
	Refocusing on Planning	12.94	3.63	13.03	3.89	−0.33	0.744	13.84	3.5	−1.33	0.183
	Positive Reapraisial	12.45	3.89	12.46	4.07	−0.03	0.972	14.56	3.9	−2.91	0.003
	Putting into perspective	12.27	3.82	11.64	3.91	2.24	0.01	11.44	2.55	1.19	0.232
	Catastrophizing	8.66	3.6	6.05	2.43	12.68	< 0.001	–	–	–	–
	Blaming others	8.84	3.53	6.38	2.69	11.43	< 0.001	–	–	–	–
	Adaptive Strategies Tot.	59.49	13.29	–	–	–	–	–	–	–	–
	Non-adaptive Strategies Tot.	41.97	10.66	–	–	–	–	–	–	–	–
		*N* = 279		*N* = 73^e^		t	*p*	mean	sd	t	*p*
		mean	sd	mean	sd						
BES	Cognitive	32.39	3.86	37.62	3.46	−10.52	< 0.001	–	–	–	–
	Affective	39.81	7.37	37.49	3.39	2.61	0.01	–	–	–	–
	Total	72.21	9.82	75.11	10.2	−2.22	0.03	–	–	–	–

Means, standard deviation (SD) and comparisons (coefficient and *p* value) between ADHD and control samples derived from five independent studies for Emotion Regulation Scale (ERS), Cognitive Emotion Regulation Questionnaire (CERQ) and Basic Empathy Scale (BES)

^a^Nock et al. 2008; ^b^Lannoy et al. 2014; ^c^Garnefski et al. 2007; ^d^ Daros et al. 2018; ^e^ Euler et al. 2017

### Cognitive emotion regulation questionnaire

ADHD patients showed higher scores for adaptive cognitive emotion regulation strategies (β = − 6.44; *p* < 0.001 and β = − 5.32; *p* < 0.05), and lower scores for non-adaptive strategies (β = 4.04; *p* < 0.01 and β = 6.62; *p* < 0.001) than BPD and ADHD+BPD patients respectively. BPD and BPD + ADHD groups did not differ. The subscale scores showed an overall similar pattern, with exception of the ‘acceptance’ (adaptive) and ‘rumination’ (non-adaptive) subscales where all three groups showed comparable scores. ADHD patients had similar scores as BPD patients in the ‘blaming others’ subscale but were still significantly lower than BPD + ADHD patients (Table 2).

Our ADHD patients showed poorer cognitive emotions regulation strategies as indicated by higher levels of all non-adaptive cognitive strategies than 611 adults from the general population control group (*p* < 0.0001 for all non-adaptive strategies, t-values ranging from 9.12 to 14.19) [47]. Results pertaining to the adaptive cognitive strategy ‘acceptance’ were less consistent across samples, with overall similar results as controls (Table 3).

### Basic empathy scale

The three groups did not differ on total or on cognitive or affective subscale scores, with the exception of lower cognitive empathy in BPD + ADHD than in ADHD patients (β = − 1.35; *p* < 0.05) (Table 2).

Compared to a community sample of adolescents [45, 48] (who are supposed to have lower empathy than adults), our ADHD patients had significantly lower total and cognitive empathy (72.21 (SD = 9.82) vs. 75.11 (SD = 10.20) t = − 2.22; *p* = 0.03 and 32.39 (SD = 3.86) vs 37.62 (SD = 3.46) t = − 10.52; *p* < 0.001 respectively) but higher affective empathy (39.81 (SD = 7.37) vs. 37.49 (SD = 3.39) t = 2.61; *p* = 0.01) (Table 3).

### Association with symptomatology

#### ADHD symptomatology (ASRSv1.1)

We constructed a model adding ASRSv1.1 score as predictor of the ERS, CERQ, and BES scores, analyzing only ADHD and ADHD+BPD patients. We found that the total ASRSv1.1 score was positively associated with ERS total score (β = 0.74, *p* < 0.001) and all the subscales (Sensitivity: β = 0.38; *p* < 0.001; Arousal/Intensity: β = 0.26; *p* < 0.001; and Persistence: β = 0.13; *p* < 0.001). This association was also true when looking at attentional or hyperactive/impulsive symptoms (data not shown).

ASRSv1.1 total score was also significantly associated with higher score on non-adaptive cognitive strategies total score (β = 0.24, *p* < 0.001) and with each of the non-adaptive subscales (data not shown). It was not associated with adaptive strategies (β = 0.03, *p* = 0.641). The significant association was observed for attentional as well as hyperactive/impulsive symptoms.

ASRSv1.1 was significantly associated with higher empathy total score (β = 0.11, *p* < 0.05), affective subscale score (β = 0.08, *p* < 0.05), but not cognitive subscale score (β = 0.03, *p* = 0.142). The association was found only with attentional symptoms (β = 0.32, *p* = 0.001 for empathy total score).

#### BDI-II as a measure of current level of distress

When adding BDI-II total score as predictor in the model, we found that current level of distress was associated with higher ERS total scores (β = 0.72, *p* < 0.001). The difference between ADHD and BPD patients was no longer significant (β = 2.48, *p* = 0.282), however the difference between ADHD and ADHD+BPD remained significant (β = 3.29; *p* < 0.005).

BDI-II total score was also predictive of lower CERQ adaptive strategies (β = − 0.31, *p* < 0.001) and higher non-adaptive strategies (β = 0.41, *p* < 0.001). When adjusting for BDI-II, the difference between groups was no longer significant (β = − 2.66, *p* = 0.197 and β = − 1.86, *p* = 0.379 for adaptive, and β = − 1.44, *p* = 0.339 and β = 1.95, *p* = 0.208 for non-adaptive strategies, respectively for difference between ADHD and BPD and ADHD and ADHD+BPD).

BDI-II was not associated with empathy total score (β = 0,02, *p* = 0.704).

#### Association with ADHD presentation

ADHD patients with the combined presentation had higher ERS total scores (β = 6.72; *p* = 0.002) than those with the attentional presentation. There were no differences between the two presentations of ADHD on CERQ adaptive and non-adaptive subscales (β = 1.64; *p* = 0.318 and β = 0.38; *p* = 0.776) or on BES total score (β = − 0.87; *p* = 0.452).

Comparing BPD patients either to patients with ADHD combined or only attentional presentations yielded similar results as comparing BPD patients to the entire ADHD patient group on the ERS total score, BES total score and CERQ adaptive and CERQ non-adaptive score.

#### Taking into account the current depressive episode

Thirty-three (47.13%) BPD, 44 (15.77%) ADHD and 25 (41.67%) BPD + ADHD patients had a current major depressive episode. When adding current major depressive episode as a predictor in the model, we found that the difference between groups was still significant, with ADHD patients showing lower ERS total score than BPD patients (b = − 8.79; *p* = 0.001) and ADHD+BPD patients (β = − 13.33; *p* < 0.001). This was true for all the ERS subscales.

Current major depressive episode was also associated with lower CERQ adaptive strategies (β = − 5.27; *p* = 0.004) and with higher CERQ non-adaptive strategies (β = 4.63; *p* = 0.001). When adjusting for current major depressive episode, ADHD patients still showed higher CERQ adaptive strategies than BPD patients (β = 4.78; *p* = 0.021), but the difference with ADHD+BPD was no longer significant (β = 3.92; *p* = 0.064). Furthermore, with this adjustment the difference between ADHD and BPD for CERQ non-adaptive strategies was no longer significant (β = − 2.87; *p* = 0.074), but was still significant for the comparison between ADHD and ADHD+BPD (β = − 561; *p* = 0.001) with lower scores in ADHD patients.

No association between BDI-II and empathy total score was found (β = 1.67; *p* = 0.126).

## Discussion

We found that ADHD patients, although having more ED than community-based psychiatric patients or controls, had significantly better emotion regulation and more efficient emotion regulation strategies than subjects suffering from BPD, and BPD and ADHD combined. Our results indeed showed lower emotional reactivity, better use of adaptive cognitive strategies and lesser use of non-adaptive strategies in ADHD patients than in subjects of the two other groups. On the other hand, cognitive and affective empathy abilities were similar between groups. Finally, emotion regulation difficulties were associated with ADHD symptomatology (specifically intensity, persistence and reactivity, and use of non-adaptive strategies).

We found that patients with ADHD had higher scores on scales measuring ED compared to community-based psychiatric patients. Previous studies suggested a slow return to emotional baseline, increased intensity and instability of negative emotions, and a predominance of negative emotions in ADHD [3, 4, 10]. Our findings thus add to the current body of literature suggesting difficulties in regulating emotions in ADHD [24, 49]. These findings may be related to the fact that ADHD patients showed an overall higher use of non-adaptive cognitive emotion regulation strategies than healthy controls. They indeed showed similar scores on the CERQ non-adaptive ‘blaming others’ and ‘rumination’ subscales as BPD patients. Rumination was previously found to be overused by BPD patients and linked to the persistence of negative emotions [19, 50]. Our results thus suggest that, similar to BPD, poor cognitive emotion regulation strategies such as ‘rumination’ as well as others such as ‘self-blame’, ‘blaming others’ and ‘catastrophizing’ to a lesser extent, play a role in ED in ADHD [17]. Of note, our results also suggest that ADHD patients mainly differ from controls by a higher tendency to use non-adaptive cognitive strategies rather than adaptive ones, similar to BPD patients [18, 51]. Nonetheless, ADHD patients still had overall less emotion sensitivity, less emotion arousal and intensity, and less persistence of the emotion than BPD and BPD + ADHD patients. Compared to the other two patient groups, they also used more adaptive and less non-adaptive cognitive emotional regulation strategies. These results suggest that, although of importance in ADHD, emotion dysregulation is not as central as it is in BPD and may explain only a part of the difficulties found in this disorder. Nevertheless, severity of ADHD was associated with higher emotional reactivity and with higher use of non-adaptive cognitive strategies. This is in agreement with other evidence suggesting that ADHD severity is closely related to difficulties in emotion regulation [7, 8]. This is also concordant with findings in BPD, which link more use of maladaptive cognitive emotion regulation strategies with higher rates of potentially harmful behaviours and severity of the disorder [17, 18]. Thus one might think that, as in BPD, using maladaptive cognitive emotion regulation strategies (which have more short-term benefits) more often than adaptive strategies helps maintain ED, which secondarily leads to increased severity of ADHD attentional and hyperactive-impulsive symptoms [17, 20, 22, 52]. These observations stress the impact of emotional symptoms on ADHD prognosis and the value of early diagnosis to address them, moreover considering the evidence that ED symptoms could mediate the relationship between childhood ADHD and adulthood BPD symptoms [13]. In addition, knowing that ED has been associated with poor global functioning, poor prognosis, severity of ADHD, higher rate of comorbidities and persistence of ADHD in adulthood, early interventions targeting this dimension are crucially needed [1, 4, 7–9, 53]. The importance of a long-term integrative approach can be highlighted by the limited effect of medication on ED [4]. Indeed research has shown that while pharmacotherapy can improve to a certain extent these symptoms, its effect seems to be lesser than on the other well-known “dysexecutive” ADHD symptoms [4, 5, 54].

We found that ADHD as well as BPD displayed lower empathy than a sample of adolescents not suffering from psychiatric disorder. This slight deficit in empathy might contribute, as in BPD, to poorer emotion regulation capacities [14, 55]. Previous research has indeed found that ADHD patients have empathic capacities that are slightly below those of healthy controls. These poorer capacities are related to difficulties in perceiving contextual information possibly linked to deficits in directing attention on emotional signals, to deficits in reflexive functioning, and to deficits in emotional face recognition [9, 14, 23]. Thus targeting empathy, either directly or indirectly, in ADHD during treatment might help decrease ED and subsequently the severity of the disorder [56, 57]. Indeed, improving mentalizing capacities - defined as linking one’s own and other people’s actions to mental states which are thus closely related to empathy – is promising for improving emotion regulation in adults suffering from ADHD [57].

Concerning ADHD presentation, the combined type was associated with higher emotional reactivity as previously shown in other studies [2, 4]. No difference in the use of cognitive emotional regulation strategies was observed.

The hypothesis of a possible additive effect of both disorders (ADHD + BPD) was not really corroborated by our results since no significant difference between BPD and ADHD+BDP was found. However, this observation might be due to a ceiling effect as the scales we used were designed to assess emotions present in the general population and not in highly emotionally dysregulated samples such as BPD.

Part of the differences between groups was related to the level of distress as measured by the BDI-II [32]. Our results showed a high level of correlation between current distress, emotional reactivity and the use of poor cognitive emotion regulation strategies in ADHD but also in BPD. This result highlights the substantial negative impact of ED on patients’ well-being and quality of life [4, 9]. It is essential to point out the low percentage of comorbidities compared to previous research, which could be explained by our study focus being mainly on current comorbidities and not on their lifetime occurrence. They were also assessed clinically without further investigations.

Our study has several limitations. Firstly, we did not have a control population and used BPD patients as a comparison to assess ED, cognitive emotion regulation strategies and empathy in ADHD patients. We nevertheless compared our patients to samples from the general population, although not matched on age and gender, derived from others studies and we are quite confident that the assessment of emotion components in our study is a true reflection of the difficulties endured by patients suffering from BPD and ADHD. Secondarily, there was a substantial difference in the size of our three groups, the ADHD group being the largest one. This may have slightly biased our results keeping in mind that ED in ADHD subjects was the main focus of our study, the two other groups being here considered as comparison samples. Thirdly we used self-report measures to assess the different aspects of emotions. However it has previously been shown that self-report measures are reliable if used in combination with interviews, which was the case in our study [58]. Finally, ED in our sample might be better explained by current comorbid disorders, especially current major depressive episode. However, when we adjusted for current major depressive episode, ADHD was still associated with less ED and more use of adaptive cognitive strategies than BPD. Therefore the difference in ED reflected by the ERS couldn’t be totally explained by the presence of more clinical depression.

## Conclusions

Our findings support the importance of exploring facets of ED as well as cognitive emotion regulation strategies in ADHD. These may, as in BPD, be an important feature of the disorder and be associated with its severity. In general, our results showed that maladaptive cognitive emotion strategies for management of emotion, such as ‘self-blame’, ‘catastrophizing’, ‘other-blame’ and ‘rumination’ are often used by ADHD patients and should be considered in individual and group psychotherapeutic approaches offered to these patients. Furthermore, ADHD patients showed a better use of cognitive emotional regulation strategies and less emotional reactivity than BPD patients. Similarities were also found between these populations, such as the tendency of using ‘blaming others’ and the absence of a difference in cognitive empathy, possibly suggesting a common deficit in perception of self and others in emotionally triggering situations.

## Data Availability

The datasets used and/or analyzed during the current study are available from the corresponding author on reasonable request.

## Abbreviations

ADHD: Attention deficit hyperactivity disorder
ARSV-v1.1: Adult ADHD Self-Report Scale
BDI-II: Beck Depression Inventory II.
BES-A: Basic Empathy Scale
BPD: Borderline personality disorder
BSL-23: *Borderline Symptoms Check-List*
CERQ: Cognitive Emotional Regulation Questionnaire
DIVA 2.0: Diagnostic Interview for ADHD in adults
ED: Emotional dysregulation
ERS: Emotion Reactivity Scale
SCID-II: Screening Interview for Axis II disorders
SD: Standard deviation
